# COVID-19 vaccine hesitancy between family decision-makers and non-decision-makers among college teachers

**DOI:** 10.1080/07853890.2022.2162114

**Published:** 2023-01-03

**Authors:** Rong Xu, Guifeng Shi, Shuo Zheng, Tao-Hsin Tung, Meixian Zhang

**Affiliations:** a Department of Nursing, Xiamen Medical College, Xiamen, Fujian, China; b Department of Preventive Health Care, Taizhou Hospital of Zhejiang Province Affiliated to Wenzhou Medical University, Linhai, Zhejiang, China; c Department of Nursing, Weifang Nursing Vocational College, Qingzhou, Shandong, China; d Evidence-Based Medicine Center, Taizhou Hospital of Zhejiang Province Affiliated to Wenzhou Medical University, Linhai, Zhejiang, China

**Keywords:** COVID-19, vaccine hesitancy, decision-makers, college teachers, China

## Abstract

**Background:**

Teachers with high educational levels significantly impact the health-related knowledge and attitudes of young students and their family members. This study aimed to investigate the coronavirus disease 2019 (COVID-19) vaccine hesitancy and associated factors, and compare the differences between decision-makers and non-decision-makers among college teachers.

**Methods:**

A cross-sectional online survey was administered across mainland China from 4 to 7 August 2021. Overall, 251 college teachers were included using snowball sampling. A multivariable logistic regression model was applied to explore the association between decision-makers and hesitancy to receive a COVID-19 vaccine.

**Results:**

Overall, 42.2% of the teachers were hesitant to being vaccinated against COVID-19. The hesitancy rate was lower among primary decision-makers than that among non-decision-makers (34.8% vs. 60.3%, *p* < .001). Primary decision-makers were less hesitant regarding COVID-19 vaccination than non-decision-makers (OR = 0.37, 95% CI 0.20–0.70); remarkably, whereas those engaged in nursing education versus non-medical related professional education (OR = 2.67, 95% CI 1.29–5.49), and partial versus full-course vaccination recipients (OR = 4.48, 95% CI: 1.76–11.42) were more likely to be hesitant regarding COVID-19 vaccination.

**Conclusion:**

Our findings indicate that a high proportion of college teachers in China are hesitant to receiving COVID-19 vaccination, and that primary decision-makers are less likely to exhibit hesitancy to being vaccinated against COVID-19 than non-decision-makers in their family. Family decision-makers among teachers can be considered a priority for COVID-19 vaccine promotion, thereby enhancing vaccine acceptance among vulnerable populations—including older adults and children—and preventing adverse outcomes.

**KEY MESSAGES**
- **Question:** How prevalent is COVID-19 vaccine hesitancy among college teachers? Do differences exist between decision-makers and non-decision-makers?
- **Findings:** We found that a substantial proportion of college teachers are hesitant to being vaccinated against COVID-19, and that family decision-makers exhibited a lower hesitancy rate than non-decision-makers.
- **Meaning:** Our findings indicate that distinguishing between family decision-makers and non-decision-makers is necessary to facilitate vaccination promotion interventions among college teachers.

## 1. Introduction

The coronavirus disease 2019 (COVID-19) pandemic, which began in 2020, has persisted for three years. As of 6 October 2022, more than 616.95 million COVID-19 cases—including 6.53 million COVID-19 deaths—have been confirmed worldwide [1]. In response to the pandemic, vaccination against COVID-19 was highly anticipated. Globally, as of 3 October 2022, more than 12.72 billion vaccine doses have been administered, and the full primary and booster vaccination coverage rates were 63.28% and 28.81%, respectively; in China, the full vaccination coverage reached 86.81%, while the booster dose coverage rate only reached 52.76% [1]. The consecutive and repetitive outbreak waves have suggested that attempting to end the COVID-19 pandemic by achieving herd immunity is practically impossible [2]. The available vaccines’ protective effect does not entail blocking the transmission of the severe acute respiratory syndrome coronavirus 2 (SARS-CoV-2); rather, it involves mitigating severe illness and death from COVID-19 [3–5]. Nevertheless, it is now widely accepted that, like influenza, the SARS-CoV-2 virus will also coexist with humans for a long time [6]. Its variants continue emerging, and vaccine breakthrough infections occur frequently [7]. Vaccines targeting relatively stable regions of the virus—including the spike protein’s stem, which is seemingly less likely to mutate —may produce longer-lasting immunity against the shapeshifting of the viruses’ variants. Unless such vaccines are developed, full vaccination and booster shots remain essential in response to the pandemic, particularly to prevent COVID-19-related hospitalizations and deaths [8,9].

Like in the case of influenza, annual COVID-19 vaccinations will necessitate vaccine acceptance. Vaccine hesitancy is a critical obstacle to achieving high COVID-19 vaccine coverage. Vaccine hesitancy is defined as the ‘delayed acceptance or refusal of vaccination despite the availability of vaccination services.’ Although large-scale clinical trials have demonstrated the current COVID-19 vaccines’ safety and efficacy, breakthrough infections continue occurring in the real world as SARS-CoV-2 virus mutates and antibody titers decline. Consequently, some people are reluctant to receiving vaccines owing to concerns regarding their protective effectiveness.

The estimated global COVID-19 vaccination acceptance rate was 68.4% when the pandemic first emerged and vaccines were only hypothetical [10]. According to a recent systematic review and meta-analysis including studies published between 2020 and July 2021, the COVID-19 vaccine acceptance rate was only 61% [11]. These results indicate that numerous people exhibited hesitancy toward COVID-19 vaccination. Historically, vaccination acceptance rates have varied by populations and epidemic phases [12,13]. In China, several studies have investigated the acceptance and hesitancy of COVID-19 vaccination in both the general population [14,15] and some specific populations, including healthcare workers [16], factory workers [17], and college students [18,19]. However, few studies have examined the college faculty population in mainland China.

The health belief model suggests that misinformation and low confidence in vaccines precipitate under-vaccination. Therefore, identifying and addressing information gaps and misconceptions regarding COVID-19 vaccines to foster the public’s confidence in these vaccines form critical foundations for promoting acceptance and sufficient vaccination. Schools are recognized as privileged operational settings that transmit health-related knowledge and convey educational messages [20]. Health education classes in public schools—an underutilized channel for health communication—exhibit the unrealized potential to disseminate accurate medical information among the youth [21]. School teachers significantly contribute in influencing families’ decision of being vaccinated by sharing vital information regarding vaccines and their official recommendations [22]. However, schoolteachers’ intention to accept a COVID-19 vaccine has exhibited high heterogeneity in different countries [23–25]. In this study, we administered a cross-sectional online survey to investigate the COVID-19 vaccine hesitancy and associated factors among college teachers in mainland China; thereafter, we compared the differences between decision-makers and non-decision-makers.

## 2. Methods

### 2.1. Study design and population

Since June 2021, we have launched a population-based survey to examine differences in knowledge, attitude, practice, and related factors pertaining to COVID-19 vaccination among different populations including both general population in the community (such as parents, elderly, patients with chronic disease) and special professional populations (such as medical personnel, college students, teachers) [26,27]. As part of the project, this study predominantly focused college teachers’ vaccination intentions and family decision-makers’ effect on vaccine hesitancy.

From 4 to 7 August 2021, we utilized convenience and snowball sampling to recruit a sample of 251 teachers from 23 colleges across mainland China. Thereafter, we administered an anonymous cross-sectional survey using the Wenjuanxing platform (Changsha Ranxing Information Technology Co., Ltd., Hunan, China). A Quick Response code for a digital questionnaire was delivered to groups of colleagues or ‘Friends circle’ on WeChat—a function that is used to share personal photos or public website links in one’s ‘Moments’ to make them visible to one’s friends on platforms such as Twitter and Facebook. The first participant to complete the questionnaire was a nursing schoolteacher working at a medical college. Most participants completing the questionnaire subsequently were medical- or nursing-related teachers. The interviewees answered the self-administered questionnaire anonymously and voluntarily. Their participation in the survey was considered informed consent. We did not utilize a separate written informed consent form to protect participants’ anonymity. As the main content of this survey is the same as that of our previous study in other different populations, this study was approved with the same ethical approval number (K20210520) by the Ethics Committee of Taizhou Hospital, Zhejiang Province, China [26,27]. All procedures were performed according to the guidelines of our institutional ethics committee and tenets of the Declaration of Helsinki.

### 2.2. Structured questionnaires

Based on our previous studies [26–28], initially, we designed a self-administered questionnaire (Chinese version). Thereafter, the initial questionnaire was assessed and modified per the feedback obtained from the pilot population, to increase reasonableness, readability, and clarity of the formal questionnaire (Appendix 1). For the sake of understanding, we also provided the English version of the questionnaire (Appendix 2).

The questionnaire comprises the following sections: (1) An informed consent statement was provided in the questionnaire’s introductory section. (2) Basic demographic information included age, sex, educational level, total service years, profession, and technical title. (3) Personal background information comprised underlying diseases; allergic history; influenza and COVID-19 vaccination history; COVID-19 risk perception; attention to COVID-19 vaccine information; knowledge regarding COVID-19 vaccine types; confidence in COVID-19 vaccines’ safety, effectiveness, and protection period. (4) Vaccine hesitancy was assessed using one question: ‘Do you hesitate to receive the COVID-19 vaccine for yourself (whether you are vaccinated or not)?’ The following four response options were provided: very hesitant, hesitant, unhesitant, or very unhesitant. The first two options were combined to indicate hesitancy, while the remaining two were combined to indicate non-hesitancy during the final analysis. (5) Thereafter, the teachers were asked the following question: ‘Are you the primary decision-maker in your family with respect to COVID-19 vaccination?’ The following two options were provided: yes and no. All the questions were close ended, with checkboxes provided for responses.

In this study, the family decision-makers with respect to COVID-19 vaccination included not only teachers making decisions or affecting their family members’ choices, but also those deciding whether to be vaccinated—such as those who are single, living alone, or without children, as they may also potentially influence the vaccination decision-making of their parents or other loved ones.

### 2.3. Statistical analysis

The survey’s primary outcome was teachers’ hesitancy to COVID-19 vaccination. Counts and frequency distributions were presented for classified data; *χ*^2^ (chi-square) tests were utilized to compare the vaccine hesitancy rate between decision-makers and non-decision-makers. Moreover, the differences in teachers’ basic characteristics between the two groups were assessed using the chi-square test.

First, potential factors associated with teachers’ vaccine hesitancy were identified using the chi-square test. Thereafter, the variables significant at the *p* < .05 level in the univariate analyses and most common confounders—including age and sex—were entered into the multiple logistic regression model, with the odds ratio (OR) and a 95% confidence interval (CI) calculated. All data were analyzed using the IBM SPSS statistics 26.0 software (SPSS Inc., Chicago, IL, USA). A *p*-value of <.05 was considered to represent a statistically significant difference among the study populations.

## 3. Results

### 3.1. Participants’ characteristics

Our analysis included 251 college teachers aged between 23 and 59 years (mean = 37.75 years, SD = 7.28); of the participants, 79.7% (200/251) were female. Most participants (*n* = 178 [70.9%]) reported that they were the primary decision-makers pertaining to COVID-19 vaccination in their family, while 29.1% (73/251) were not decision-makers. The primary decision-makers’ proportion among males was higher than that among females (82.4% vs. 68.0%, *p* = .044). Table 1 summarizes the participants’ characteristics in primary decision-makers and non-decision-makers. A higher proportion of teachers focused on (48.9% vs. 32.9%, *p* = .02) and knew regarding (50.6% vs. 34.2%, *p* = .018) COVID-19 vaccine information in the primary decision-makers than in the non-decision-makers. Additionally, full-course vaccinations’ proportion was higher in the primary decision-makers than that in the non-decision-makers (86.0% vs. 75.3%, *p* = .04). No differences were observed in age group; educational level; service years; profession and professional titles; underlying diseases; allergic history; influenza vaccination history; COVID-19 risk perception; and confidence in COVID-19 vaccines’ safety, effectiveness, and protection period between the primary decision-makers and non-decision-makers (*p* > .05).

**Table 1.** Basic characteristics of college teachers: decision-makers versus non-decision-makers (*n* = 251).

Variables	Categories	All (*n* = 251)	Primary decision-makers (*n* = 178)	Non-decision-makers (*n* = 73)	*χ2*	*P*
Sex	Male	51 (20.3)	42 (23.6)	9 (12.3)	4.059	**.044**
	Female	200 (79.7)	136 (76.4)	64 (87.7)		
Age (years)	Less than 40	173 (68.9)	120 (67.4)	53 (72.6)	0.65	.420
	40 and above	78 (31.1)	58 (32.6)	20 (27.4)		
Education level	Undergraduate	81 (32.3)	60 (33.7)	21 (28.8)	0.578	.447
	Graduate	170 (67.7)	118 (66.3)	52 (71.2)		
Total service time (years)					1.578	.209
	<10	122 (48.6)	82 (46.1)	40 (54.8)		
	≥10	129 (51.4)	96 (53.9)	33 (45.2)		
Profession					1.831	.400
	Physician teaching	56 (22.3)	43 (24.2)	13 (17.8)		
	Nurse teaching	128 (51.0)	91 (51.1)	37 (50.7)		
	Non-medical related teaching	67 (26.7)	44 (24.7)	23 (31.5)		
Professional titles	Primary grade	50 (19.9)	33 (18.5)	17 (23.3)	0.732	.694
	Medium grade	122 (48.6)	88 (49.4)	34 (46.6)		
	Associate professor or professor	79 (31.5)	57 (32.0)	22 (30.1)		
Frequency of taking a cold (times/year)						
	0-1	123 (49.0)	89 (50.0)	34 (46.6)	0.243	.622
	≥2	128 (51.0)	89 (50.0)	39 (53.4)		
Do you suffer from chronic diseases?					0.421	.516
	Yes	14 (5.6)	11 (6.2)	3 (4.1)		
	No	237 (94.4)	167 (93.8)	70 (95.9)		
Allergic history					0.014	.906
	Yes	47 (18.7)	33 (18.5)	14 (19.2)		
	No	204 (81.3)	145 (81.5)	59 (80.8)		
History of influenza vaccination					0.003	.958
	Yes	59 (23.5)	42 (23.6)	17 (23.3)		
	No	192 (76.5)	136 (76.4)	56 (76.7)		
Risk perception of COVID-19	High	42 (16.7)	31 (17.4)	11 (15.1)	0.205	.651
	Low	209 (83.3)	147 (82.6)	62 (84.9)		
Attention to COVID-19 vaccine information					5.373	**.020**
	Yes	111 (44.2)	87 (48.9)	24 (32.9)		
	No	140 (55.8)	91 (51.1)	49 (67.1)		
Know about the COVID-19 vaccine type					5.551	**.018**
	Yes	115 (45.8)	90 (5.6)	25 (34.2)		
	No	136 (54.2)	88 (49.4)	48 (65.8)		
Confidence in safety of the COVID-19 vaccines					.004	.950
	High	223 (88.8)	158 (88.8)	65 (89.0)		
	Low	28 (11.2)	20 (11.2)	8 (11.0)		
Confidence in effectiveness of the COVID-19 vaccines					2.563	.109
	High	189 (75.3)	139 (78.1)	50 (68.5)		
	Low	62 (24.7)	39 (21.9)	23 (31.5)		
How long do you think the protective effect will last after vaccination?					1.649	.438
	6 months	141 (56.2)	101 (56.7)	40 (54.8)		
	12 months	74 (29.5)	49 (27.5)	25 (34.2)		
	≥24 months	36 (14.3)	28 (15.7)	8 (11.0)		
How many doses of the COVID-19 vaccine have you received so far?					6.458	**.040**
	0	14 (5.6)	6 (3.4)	8 (11.0)		
	1	29 (11.6)	19 (10.7)	10 (13.7)		
	2	208 (82.9)	153 (86.0)	55 (75.3)		

### 3.2. Vaccine hesitancy prevalence in college teachers separated by decision-makers or non-decision-makers

**Figure 1.**
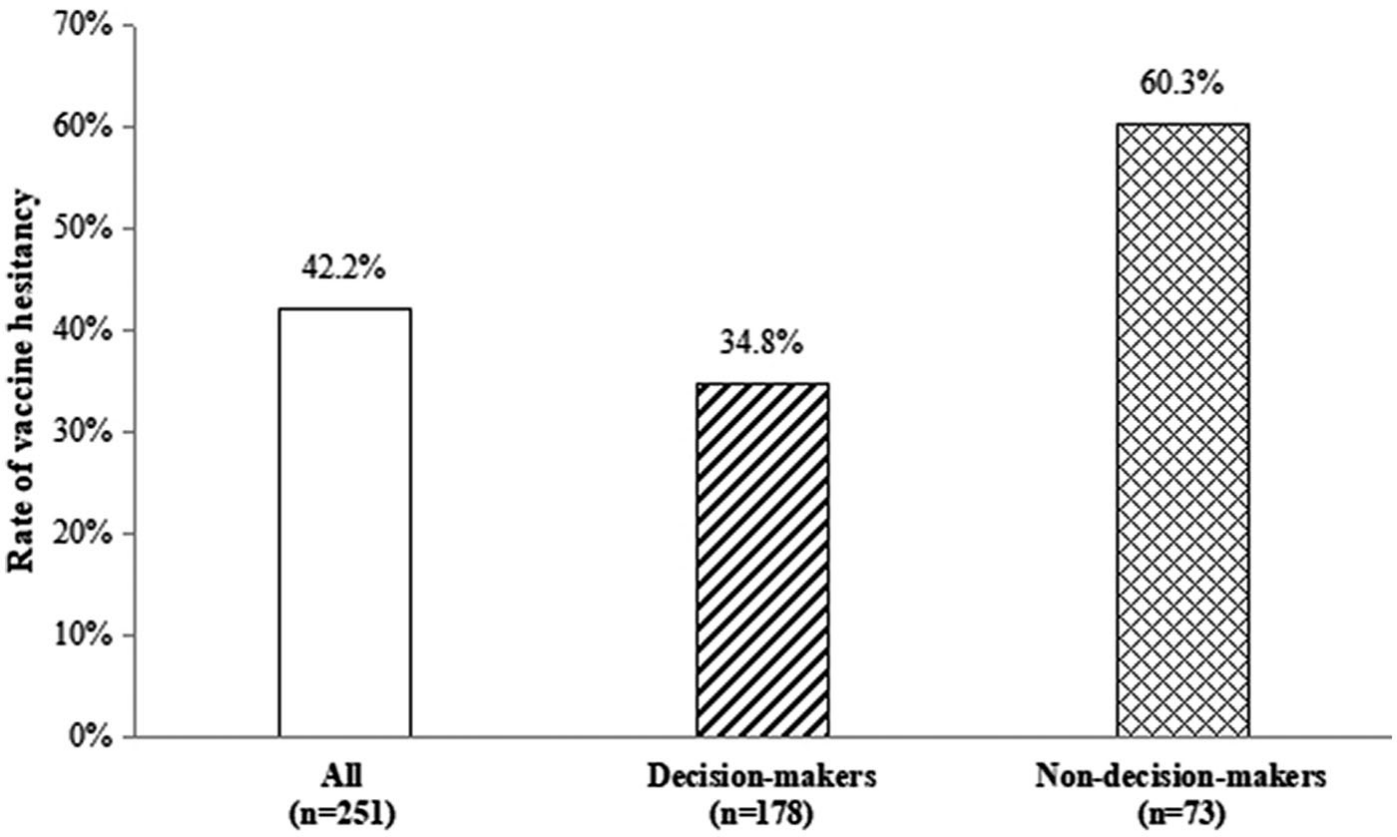
Vaccine hesitancy among college teachers: decision-makers versus non-decision-makers.

Overall, 42.2% (106/251) of the college teachers reported that they were hesitant regarding COVID-19 vaccination, with 34.8% (62/178) of the primary decision-makers and 60.3% (44/73) of non-decision-makers reporting vaccine hesitancy (*p* < .001; Figure 1).

### 3.3. Vaccine hesitancy’s influencing factors

First, we compared participants’ basic characteristics between hesitancy—and no hesitancy—to be vaccinated against COVID-19, and identify potential factors influencing vaccine hesitancy in the univariate analyses (Table 2). Thereafter, a multiple logistic regression analysis was performed, the results of which are presented in Table 3. The risk factors for vaccine hesitancy were as follows: being engaged in nursing education (OR = 2.67, 95% CI 1.29–5.49) and vaccination dose (vaccinated 1 dose vs. vaccinated 2 doses: OR = 4.48, 95% CI 1.76–11.42; unvaccinated vs. vaccinated 2 doses: OR = 3.30, 95% CI 0.89–12.24). By contrast, the teacher being the primary decision-maker in the family with respect to COVID-19 vaccination reduced the likelihood of vaccine hesitancy (OR = 0.37, 95% CI 0.20–0.70).

**Table 2.** Univariate analysis of COVID-19 vaccine hesitancy among college teachers (*n* = 251).

Variables	Categories	All (*n* = 251)	No hesitancy (*n* = 145)	Hesitancy (*n* = 106)	*χ2*	*P*
Sex	Male	51 (2.3)	35 (24.1)	16 (15.1)	3.093	.079
	Female	200 (79.7)	110 (75.9)	90 (84.9)		
Age (years)	23-39	173 (68.9)	102 (7.3)	71 (67.0)	.323	.570
	40-59	78 (31.1)	43 (29.7)	35 (33.0)		
Education level	Undergraduate	81 (32.3)	54 (37.2)	27 (25.5)	3.881	**.049**
	Graduate	170 (67.7)	91 (62.8)	79 (74.5)		
Profession					6.464	**.039**
	Physician teaching	56 (22.3)	37 (25.5)	19 (17.9)		
	Nurse teaching	128 (51.0)	64 (44.1)	64 (6.4)		
	Non-medical related teaching	67 (26.7)	44 (3.3)	23 (21.7)		
Total service time (years)					.015	.903
	<10	122 (48.6)	70 (48.3)	52 (49.1)		
	≥10	129 (51.4)	75 (51.7)	54 (5.9)		
Professional titles	Primary grade	50 (19.9)	32 (22.1)	18 (17.0)	1.566	.457
	Medium grade	122 (48.6)	66 (45.5)	56 (52.8)		
	Associate professor or professor	79 (31.5)	47 (32.4)	32 (3.2)		
Frequency of taking a cold (times/year)					.247	.619
	0-1	123 (49.0)	73 (5.3)	50 (47.2)		
	≥2	128 (51.0)	72 (49.7)	56 (52.8)		
Do you suffer from chronic diseases?					1.351	.245
	Yes	14 (5.6)	6 (4.1)	8 (7.5)		
	No	237 (94.4)	139 (95.9)	98 (92.5)		
Allergic history					.002	.960
	Yes	47 (18.7)	27 (18.6)	20 (18.9)		
	No	204 (81.3)	118 (81.4)	86 (81.1)		
History of influenza vaccination					.772	.379
	Yes	59 (23.5)	37 (25.5)	22 (2.8)		
	No	192 (76.5)	108 (74.5)	84 (79.2)		
Risk perception of COVID-19						
	High	42 (16.7)	26 (17.9)	16 (15.1)	.354	.552
	Low	209 (83.3)	119 (82.1)	90 (84.9)		
Attention to COVID-19 vaccine information					1.574	.210
	Very	111 (44.2)	69 (47.6)	42 (39.6)		
	Quite	140 (55.8)	76 (52.4)	64 (6.4)		
Know about the COVID-19 vaccine type					7.344	**.007**
	Yes	115 (45.8)	77 (53.1)	38 (35.8)		
	No	136 (54.2)	68 (46.9)	68 (64.2)		
Confidence in safety of the COVID-19 vaccines					4.413	**.036**
	High	223 (88.8)	134 (92.4)	89 (84.0)		
	Low	28 (11.2)	11 (7.6)	17 (16.0)		
Confidence in effectiveness of the COVID-19 vaccines					4.080	**.043**
	High	189 (75.3)	116 (8.0)	73 (68.9)		
	Low	62 (24.7)	29 (2.0)	33 (31.1)		
How long do you think the protective effect will last after vaccination?					8.559	**.014**
	6 months	141 (56.2)	71 (49.0)	70 (66.0)		
	12 months	74 (29.5)	47 (32.4)	27 (25.5)		
	≥24 months	36 (14.3)	27 (18.6)	9 (8.5)		
How many doses of the COVID-19 vaccine have you received so far?					18.97	**<.001**
	0	14 (5.6)	4 (2.8)	10 (9.4)		
	1	29 (11.6)	8 (5.5)	21 (19.8)		
	2	208 (82.9)	133 (91.7)	75 (7.8)		
Are you the primary decision-maker in your family regarding the COVID-19 vaccine?					13.736	**<.001**
	Yes	178 (70.9)	116 (80.0)	62 (58.5)		
	No	73 (29.1)	29 (20.0)	44 (41.5)		

**Table 3.** Multiple logistic regression analysis of factors associated with teachers’ hesitancy to receive COVID-19 vaccine (*n* = 251).

Variables	Categories	*p*	*OR* (95%*CI*)
Sex	Male vs. female	.712	1.16 (0.53–2.53)
Age group (years)	40–59 vs. 23–39	.193	1.52 (0.81–2.84)
Education level	Graduate vs. undergraduate	.105	1.70 (0.90–3.23)
Profession	Non-medical related majors		1.00
	Physician training	.773	1.14 (0.47–2.73)
	Nurse training	**.008**	2.67 (1.29–5.49)
Know about the COVID-19 vaccine type	Yes vs. no	.097	0.61 (0.34–1.09)
Confidence in safety of the COVID-19 vaccines	High vs. low	.232	0.57 (0.23–1.43)
Confidence in effectiveness of the COVID-19 vaccines	High vs. low	.423	0.76 (0.39–1.48)
How long do you think the protective effect will last after vaccination?	6 months		1.00
	12 months	.149	0.62 (0.32–1.19)
	≥24 months	.183	0.54 (0.22–1.34)
How many doses of the COVID-19 vaccine have you received so far?	Vaccinated 2 doses		1.00
	Vaccinated 1 dose	**.002**	4.48 (1.76–11.42)
	Unvaccinated	.074	3.30 (0.89–12.24)
Are you the primary decision-maker in your family regarding the COVID-19 vaccine?	Yes vs. no	**.003**	0.37 (0.20–0.70)

## 4. Discussion

### 4.1. Implications of vaccine hesitancy among college teachers

The adverse effects of COVID-19 on people’s lives, production and livelihoods are ongoing. Vaccination to expand immunization coverage is indispensable to control the spread of the pandemic. Vaccine hesitancy encompasses varying attitudes, ranging from a positive demand for a specific vaccine to a complete rejection of all vaccines. It hinders the achievement of high immunization coverage and community immunization against the infection, thereby posing a serious threat to human health worldwide.

A growing number of studies have discussed the heterogeneity of vaccine hesitancy, which is related to personal, social, cultural, and political factors [29,30]. Most evidence suggested that a lower educational level can be a potential barrier to vaccine acceptance in some settings [31,32]. However, no consensus has been reached on this association in other studies—some of which report either a contrasting [15,33] or insignificant [34] association. In this study, the educational level (graduate vs. undergraduate) of college teachers had no significant effect on their hesitancy to receive the COVID-19 vaccination. This result can be explained by the relatively concentrated distribution of educational levels of the college faculty included in this study—including graduates or undergraduates—that are relatively higher than that of the general population.

As a high education priority population, teachers can ensure continuity in education, minimize social instability, disseminate sound health knowledge, and promote the health conception development of the youth amid the COVID-19 pandemic. This study estimated that the rate of vaccine hesitancy in college teachers was 42.2%, which is much higher than that in the general adult population during the same period (8.4%) [35]. To the best of our knowledge, there have been only a few reports on COVID-19 vaccine hesitancy among teachers. We reviewed the literature on teachers’ intention to receive the COVID-19 vaccine; as shown in Table 4, their willingness to get vaccinated ranged from 31.7% to 95.6% across studies from Taizhou in China [19], Greece [23], Ethiopia [25,36,37], Italy [38], Ghana [39,40], Germany [41], Canada [24], and Taiwan [42].

**Table 4.** Estimates of teachers’ intention to accept COVID-19 vaccines.

First author	Study design	Study period	Study sample	Country	Prevalence
Chen Y, et al. [19]	Cross-sectional	June 2021	167 teachers	Taizhou, China	31.7%
Gkentzi D, et al. [23]	Cross-sectional	June to August 2020	399 teachers	Greece	38.1%
Racey CS, et al. [24]	Cross-sectional	August to November 2020	5076 teachers	Canada	89.7%
Shitu K, et al. [25]	School-based cross-sectional study	December 2020 to February 2021	301 school teachers, age range of 21 to 64 years	Northwest Ethiopia	40.8%
Zewude B, et al. [36]	institution-based cross-sectional study	March 2021	319 primary and secondary school teachers, bank employees, and university instructors	Southern Ethiopia	46.1%
Asmare Adella G. [37]	institution-based cross-sectional study	1 June 2021 to 30 July 2021	418 teachers	Southwest Ethiopia	79.7% had positive attitudes
La Vecchia C, et al. [38]	cross-sectional survey	16–28 September 2020	1055 individuals (546 women, 509 men) aged 15–85 years	Italy	51.6%
Amo-Adjei J, et al. [39]	Sequential mixed method design	April and May 2021	52 teachers	Ghana	73.1%
Dubik SD [40]	Cross-sectional	April to September 2021	421 teachers	Northern Ghana	49%, 63%, and 11% before rollout, after rollout, and actual uptake, respectively
Weinert S, et al. [41]	Cross-sectional	2 March and 30 April 2021	6753 teachers	Germany	77%
Duong TV, et al. [42]	Cross-sectional	23 June to 16 July 2021	387 School Principals	Taiwan, China	95.6%

Teachers often live and work in crowded environments, and are thus at high risk of infection. According to the Theory of Reasoned Action (TRA), due to the authoritative nature of their profession, teachers’ choices may influence students’ decision-making processes through attitudes and subjective norms. Teachers can reinforce students’ concept of herd immunity, explain the risk-benefit relationship of vaccination, and raise their awareness of misinformation, so as to reduce the hesitancy of students and their families to get vaccinated. Thus, because of their role in disseminating health knowledge and health literacy, the immunization coverage rate of college teachers must be increased.

### 4.2. Reason for high levels of vaccine hesitancy among teachers

The relatively high rate of vaccine hesitancy among college teachers may be related to their professional characteristics. College teachers are particularly important public health actors not only among their students but also among parents and the community [43]. Compared to other professionals, college teachers with higher education may be more knowledgeable about the safety and efficacy of the vaccines. They typically pay more attention to vaccine-related news and the perceived reduced effectiveness of vaccination. Absence of confidence and trust in the safety and effectiveness of the COVID-19 vaccines, along with the inadequate assessment of the risk-benefit ratio of getting vaccinated, may account for their high levels of vaccine hesitancy among college teachers [44]. In this study, 106 college teachers—especially those in nursing education—were found to be hesitant to receive COVID-19 vaccines. The main reason for their vaccine hesitancy was fear of the safety of the vaccine (83.0%), followed by concerns about the effectiveness of the vaccine (44.3%) and personal physical factors (34.0%). According to the risk-averse theory, people are more likely to receive a vaccine when its perceived benefits and/or the perceived risks of the infectious disease outweigh the perceived risks of the vaccine [45]. Additionally, concerns about the adverse effects of the COVID-19 vaccine mediated the relationship between attitudes toward the effectiveness of vaccines and proactively taking vaccines for one’s family [46]. Therefore, conducting a health awareness campaign among college teachers about the vaccine’s safety and the risk-benefit relationship of vaccination is highly recommended.

### 4.3. Vaccine hesitancy and family decision-making

The decision-making process of medical intervention usually involves consensus among medical service providers, recipients, and their family members [47]. Several studies have recommended the shared clinical decision-making process to enhance vaccination rates [48], including the influenza [49], pneumonia [50], and human papillomavirus [51] vaccines. An individual may be expected to assume the role of the family decision-maker when interdependent family members participate in the decision-making process [52,53]. In this study, we hypothesized that college teachers are likely to be the primary decision-makers regarding the vaccination of their families. This is the first study to have examined the influence of the decision-making competence on vaccine hesitancy among college teachers; we found that the proportion of vaccine hesitancy among primary decision-makers was significantly lower than that among non-decision-makers (34.8% vs. 60.3%). This result is in line with our previous findings that the family decision-makers were more willing to pay for COVID-19 vaccines in a community of Taizhou, China [28]. Decision-making regarding proactively taking the COVID-19 vaccine for family may be modulated by concerns about the safety and effectiveness of the vaccine [46]; considering this, health promotion of the risk-benefit relationship of vaccination for family decision-makers must be urgently undertaken. Interventions aimed at primary decision-makers would not only change their individual attitudes but also influence the subjective norms of their family members, relatives, and friends. This would help influence their behavioral intentions and decision-making, and thereby achieve a multiplier effect with half the effort. Thus, it is of great importance to conduct interventions for primary decision-makers among teachers to increase the COVID-19 vaccination coverage rate.

Regarding decision-makers in the family, previous studies have illustrated that mothers often take on this role for their children’s healthcare, and that men are more likely than women to be legally designated as decision-makers for a major medical treatment decision that has life-or-death implications. In addition, spouses are often identified as the primary decision-maker. Given the close relationships among family members, it is necessary for public health policymakers to develop family-based vaccine promotion programs, in which family members must be expected to reach a consensus in the decision-making process.

### 4.4. Limitations

This study was the first to estimate the vaccine hesitancy of college teachers, categorizing them as primary decision-makers and non-decision-makers. However, the methodological considerations in this study had several weaknesses. First, based on a cross-sectional design, our estimates were evaluated at a single time point; thus we could not evaluate long-term vaccine hesitancy, because intention to vaccinate may change in response to the dynamic nature of the pandemic. Second, a small convenient sample was enrolled *via* snowball sampling, and the online survey may limit the representativeness of the study sample. Third, vaccine hesitancy was not measured using a formal scale but through a one-item self-report question. Similarly, the role of the family decision-maker in COVID-19 vaccination was also assessed using only one question. Therefore, it is difficult to verify the reliability and validity of the study findings. Future research needs to assess more precisely and clearly distinguish primary decision-makers from non-decision-makers in the families. Fourth, volunteer bias and the Hawthorne effect also need to be considered, as the study population was voluntarily participating in the survey. Finally, it was difficult to obtain detailed information regarding socioeconomic status and other unknown confounding factors, all of which may be risk factors contributing to vaccine hesitancy.

## 5. Conclusion

Vaccination does not stop the spread and transmission of the SARS-CoV-2 virus; however, it will prevent serious outcomes in those who are vulnerable. Therefore, vaccination remains indispensable to control the pandemic, especially for susceptible groups such as elderly people and children, who need to be actively vaccinated. Our study demonstrated a substantially high proportion of COVID-19 vaccine hesitancy among college teachers during the pandemic. Primary decision-makers in families are less likely to be hesitant to receive the COVID-19 vaccine than non-decision-makers in their family. Considering the influence of being a college faculty member and the family decision-making role, this study’s findings imply that teachers who are family decision-makers should be prioritized for vaccine promotion.

## Data Availability

The raw data are available from the corresponding author upon reasonable request. The authors have no proprietary interest in any aspect of this study. There was no additional financial support from public or private sources.

## Appendix 1. Questionnaire assessing knowledge, attitude, and practice pertaining to COVID-19 vaccination among college teachers (Chinese version)

高校教师新冠疫苗相关知识、态度和意愿调查

尊敬的老师：您好！

抗击疫情, 人人有责。随着新冠疫苗接种的快速推进, 截至8月3日全国已累计接种超过17亿剂次。但病毒不断变异, 疫情反复, 群体免疫屏障的建立依然任重而道远。基于此, 我们设计了这份新冠疫苗接种相关知识、态度和意愿的调查问卷, 请您根据实际情况作答。 本问卷采用匿名方式, 您填写的信息会严格保密。感谢您的支持和参与！

**A 基本信息**
**A1 您的性别：**□男  □女
**A2 您的年龄:**  □ □周岁
**A3 您的文化程度：** □大专  □本科  □硕士  □博士
**A4 您目前所从事的专业?**
   □临床医学  □护理学  □药学  □医技专业
   □基础医学  □公共卫生/预防医学  □中医学  □其他 _________________
**A5 您目前的职称：** □初级  □中级  □副高级  □正高级
**A6 您的工作年限：** □<5年  □5 ∼ 9年  □10 ∼ 14年  □15 ∼ 19年  □≥20年
**A7 您平时感冒的频率：** □0-1次/年  □2-3次/年  □4-5次/年  □多于5次/年
**A8 您是否患有下列慢性病?[多选题]**
   □高血压
   □糖尿病
   □心脑血管疾病（脑梗、心梗、心衰等）
   □慢性呼吸系统疾病（慢性支气管炎、哮喘等）
   □慢性肾脏疾病
   □慢性肝病
   □癌症（恶性肿瘤）
   □以上均无
**A9 既往是否有食物或药物过敏史：** □否  □是
**A10 既往是否接种过流感疫苗：** □否  □是
**B 新冠疫苗接种相关认知、态度和行为**
**B1 您认为您感染新冠病毒的风险程度：** □非常高  □高 □一般  □低  □非常低
**B2 近半年来, 您关注新冠疫苗信息的程度：**
   □非常关注 □比较关注 □偶尔关注 □没有关注
**B3 您对新冠疫苗不同种类的了解程度：**
   □非常了解 □了解 □一般 □不了解  □非常不了解
**B4 您觉得目前新冠疫苗的安全程度：**
   □非常安全  □安全  □一般  □不安全  □非常不安全
**B5 您认为疫苗对新冠病毒感染的预防作用有多大?**
   □作用很大  □作用较大  □有点作用  □没有作用
**B6 您认为接种新冠疫苗后, 保护效果能维持多长时间?**
   □6个月  □12个月  □24个月及以上
**B7 目前您接种了几针新冠疫苗?** □0  □1  □2
**B8 您是否对接种新冠疫苗存在过犹豫（不管您是否接种了新冠疫苗）?**
   □非常犹豫  □犹豫  □不犹豫  □非常不犹豫
**B9 您犹豫的原因有哪些?**
   □觉得没必要接种
   □担心疫苗安全性
   □怀疑疫苗的有效性
   □自身身体原因
   □想先观望别人接种的情况
   □觉得接种太麻烦
   □其他
**B10 在接种新冠疫苗方面, 您是家庭的主要决策者吗?** □是  □否

## Appendix 2. Questionnaire assessing knowledge, attitude, and practice pertaining to COVID-19 vaccination among college teachers (English version)

**Questionnaire assessing knowledge, attitude, and practice pertaining to COVID-19 vaccination among college teachers**

Dear teachers: Fighting the COVID-19 pandemic is everyone’s responsibility. As of August 3, more than 1.7 billion doses of COVID-19 vaccines have been administered nationwide. However, the virus continues mutating, and the pandemic keeps recurring. Achieving herd immunity is still a distant goal. This questionnaire examines knowledge, attitude, and practice pertaining to COVID-19 vaccination among college teachers. This questionnaire is anonymous, and the data collected herein will be only used for statistical analysis. This study has been approved by the appropriate ethics committee. Your participation in this survey is completely voluntary. Thank you for your support and participation.

• Basic Information

1. **Sex:**  □ 1 = male  □ 2 = female
2. **Date of Birth:**  □  □  □  □ year □  □ month □  □ day
3. **Education Level:**
    □ 1 = Junior College or Below  □ 2 = Undergraduate  □ 3 = Graduate
4. **What major are you currently engaged in:**
    □ 1 = Clinical medicine  □ 2 = Nursing  □ 3 = Pharmacy
    □ 4 = Medical technology  □ 5 = Basic medicine  □ 6 = Preventive medicine
    □ 7 = Chinese medicine  □ 8 = Others
5. **Professional titles:**
    □ 1 = Internship or primary  □ 2 = Medium
    □ 3 = Associate professor  □ 4 = Professor
6. **Total service time (years):**  □  <5  □  5–9  □ 10–14  □ 15–19  □ ≥20
7. **How often do you catch a cold?**
    □ 0–1 time/year  □ 2–3 times/year
    □ 4–5 times/year  □ More than 5 times/year
8. **Did you have any underlying diseases prior to vaccination against COVID-19?**
    □ 0 = None
    □ 1 = Hypertension
    □ 2 = Diabetes
    □ 3 = Cardiovascular/ cerebrovascular disease (such as cerebral infarction, myocardial infarction, or heart failure).
    □ 4 = Chronic respiratory diseases (such as bronchitis or asthma)
    □ 5 = Chronic kidney disease
    □ 6 = Chronic liver disease
    □ 7 = Cancer/ malignant tumors
    □ = Other diseases
9. **Have you ever had an allergic reaction to drugs, food, pollen, or similar substances?**
    □ 1 = No  □ 2 = Yes
10. **Have you ever been vaccinated against influenza?**
    □ 1 = No  □ 2 = Yes

• Knowledge, Attitude, and Practice related to COVID-19 Vaccination

1. How do you perceive the risk of the SARS-CoV-2?
    □ 1 = Very high  □ 2 = High  □ 3 = Moderate  □ 4 = Low  □ 5 = Very low
2. Have you been following news regarding the COVID-19 vaccine for the last six months?
    □ 1 = Always  □ 2 = Often  □ 3 = Occasionally  □ 4 = Never
3. How much do you know about type of COVID-19 vaccines?
    □ 1 = Highly informed  □ 2 = Well-informed  □ 3 = Only general knowledge
    □ 4 = Not very informed  □ 5 = Very little informed
4. Do you have confidence in the safety of COVID-19 vaccines?
    □ 1 = Very high  □ 2 = High  □ 3 = Moderate  □ 4 = Low  □ 5 = Very low
5. Do you have confidence in the effectiveness of COVID-19 vaccines?
    □ 1 = Very high  □ 2 = High  □ 3 = Moderate  □ 4 = Low  □ 5 = Very low
6. How long do you think the protective effect against COVID-19 will last after vaccination?
    □ 1 = 6 months  □ 2 = 12 months  □ 3 = 24 months and above
7. How many doses of the COVID-19 vaccine have you been vaccinated with?
    □ 0 = Unvaccinated  □ 1 = Vaccinated one dose  □ 2 = Vaccinated two doses
8. Do you hesitate to receive the COVID-19 vaccine for yourself (Whether you are vaccinated or not)?
    □ 1 = Very hesitant  □ 2 = Hesitant  □ 3 = Unhesitant  □ 4 = Very unhesitant
9. What are the main reasons that you are hesitant to receive vaccines against COVID-19? [Multiple choice]
    □ 1 = I do not believe that getting vaccinated is necessary.
    □ 2 = I am worried regarding the side effects of the vaccine.
    □ 3 = I doubt the effectiveness of the vaccine.
    □ 4 = I am unsure whether I can receive the vaccine because of my underlying diseases.
    □ 5 = I want to wait and see what happens when someone else gets vaccinated.
    □ 6 = I experienced difficulties when making an appointment, and waiting was an excessive inconvenience.
    □ 7 = Others
10. Are you the primary decision-maker in your family with respect to COVID-19 vaccination?
    □ 1 = Yes  □ 2 = No

––––––––––––––––––––––-The End––––––––––––––––––––––––
